# Perceptions and beliefs of general practitioners on their role in the cancer screening programmes in the Netherlands: a mixed-methods study

**DOI:** 10.1186/s12875-024-02394-5

**Published:** 2024-04-24

**Authors:** Thomas H.G. Bongaerts, Frederike L. Büchner, Vera Nierkens, Matty R. Crone, Onno R. Guicherit, Mattijs E. Numans

**Affiliations:** 1 Health Campus The Hague, Leiden University Medical Center, The Hague, the Netherlands; 2 Department of Public Health and Primary Care, Leiden University Medical Center, Leiden, the Netherlands; 3 Department of Health Promotion, Maastricht University, Maastricht, the Netherlands; 4 University Cancer Center Leiden – The Hague, Haaglanden Medical Center, The Hague, the Netherlands

**Keywords:** Cancer screening, Participation, General practitioner, Primary care, Netherlands

## Abstract

**Background:**

In the Netherlands, population-based cancer screening programmes (CSPs) are organized aiming at cervical, breast and colorectal cancer. For a CSP to be effective, high participation rates are essential; however, there is an alarming downward trend, including wide regional variation in screening uptake. General practitioner (GP) involvement can have a stimulating effect on screening participation. Current GP involvement is however, limited, varies between the programmes and has changed over time. Unexplored is what GPs think of their role(s) in the CSPs. The aim of this study was therefore to map the perceptions and beliefs of GPs regarding their current and future role in the Dutch CSPs.

**Methods:**

A mixed-methods sequential explanatory study was conducted in the Leiden/The Hague area of the Netherlands, between the end of 2021 and 2022. A questionnaire was developed and distributed among 110 GPs. The aggregated results obtained from the questionnaires served as starting points for conducting semi-structured interviews, with purposefully selected GPs. With this sequential approach we aimed to further enhance the understanding of the questionnaire data, and delved into the topics that emerged from the questionnaire responses.

**Results:**

In total, 46 GPs completed the online questionnaire (response rate 42%). Subsequent five semi-structured comprehensive interviews were conducted. GPs indicated that they frequently encounter the CSP in their daily practice and consider it important. They also emphasised it is important that GPs remain closely involved with the CSPs in the future. Nevertheless, GPs also repeatedly mentioned that they are not eager to take on more logistical/organizational tasks. They are however willing to empower CSPs in a positive manner.

**Conclusion:**

GPs were generally positive about the CSPs and their current role within these programmes. Nevertheless, several options have been proposed to improve the CSPs, especially to increase screening uptake for populations in a socioeconomically disadvantaged position. Since it is of utmost importance to screen those who are most at risk of developing the screening-specific tumours, efforts should be made to achieve this goal.

**Supplementary Information:**

The online version contains supplementary material available at 10.1186/s12875-024-02394-5.

## Introduction

The Dutch government invests considerable budgets, time and effort in hosting three population-based cancer screening programmes (CSPs), aiming at cervical, breast and colorectal cancer (CRC). The goal of these screening programmes (SPs) is to detect cancer in an early or precursor stage. On average, this approach leads to a better prognosis, as well as fewer and less severe side effects of treatment [1–3]. The screening tests of the CSPs are offered free of charge by the Dutch government to all citizens of a specific age and gender. The National Institute for Public Health and the Environment (RIVM) and the national screening organisation (Bevolkingsonderzoek Nederland) are in charge of organizing and coordinating these programmes [4, 5]. Participation is voluntary and monitored yearly by the RIVM [6–8]. Although the three CSPs exhibit many similarities, each CSP has its unique procedures and organization, mainly due to differences in screening methods (see Table 1).

**Table 1:** Key characteristics of the population-based cancer screening programmes of the Netherlands

	CC-SP	BC-SP	CRC-SP
**Since** (year)	1979 (pilots from 1976)	1990 (pilots from 1984)	2014 (fully operational since 2019)
**Population**			
Age boundaries	30–60	50–75	55–75
Sex	F	F	F & M
**Interval** (years)	5	2	2
**Screening test**	HPV-test, if HPV positive then cytology (Pap-smear)	Mammography (bilateral)	Faecal Immunochemical Test (FIT)
**General practitioner involvement**	Performing pap-smear, discuss outcome, hospital referral^a^	Discuss outcome, hospital referral^b^	None^c^; discuss outcome
**Screening outcome**	HPV absent, present or unclear (re-testing). When applicable Pap-classification and HPV-typology.	Abnormality absent, abnormality present, not enough information (BI-RADS-code 0–5)	Negative (no examination needed), positive (examination needed), unclear (re-testing)
**Financing**			
Invitation, screening test(s) and analyses	Dutch government		
Secondary test(s) and treatment	Standard healthcare, hence depending on one’s individual insurance policy		

*CC *Cervical Cancer, *BC *Breast Cancer, *CRC* Colorectal Cancer, *SP* Screening programme, *F*  Female, *M* Male, *HPV* Human Papillomavirus

^a^From 2017 onward, women can opt to receive a self-sampling test (after being invited). The outcome of the self-sampling test is not automatically shared with the GP due to privacy legislation. Outcomes will only be shared with the GP if it is explicitly stated that the GP is allowed to receive this information. Hence, the GP no longer plays an essential role in this CSP. If HVP is detected, women are recommended to contact their GP to have a smear test taken at the GP practice

^b^In cases where no abnormalities are detected, the GP will not be involved

^c^Since 2017, the GP no longer automatically receives the outcome of a FIT. Outcomes will only be shared with the GP if it is explicitly stated that the GP is allowed to receive this information. After a positive FIT patients are encouraged to seek contact with their GP

High participation rates are essential for a CSP to be effective. According to the World Health Organization (WHO), at least 70% of the target population should be screened in order to be beneficial at the population level [9–11]. Throughout Europe participation in CSPs varies substantially, yet the Netherlands has always been known for its high screening attendance and adherence [12]. The most recent nationally available attendance rates – registered before the COVID-19 pandemic – were 56.0%, 76.0% and 71.8% for the SPs aimed at cervical, breast and CRC, respectively [6–8]. Although the attendance rates of two programmes are above the recommended rate from WHO, there is an alarming downward trend and wide regional variation in screening uptake [13]. In 2010, the uptake rates of the CSPs for cervical and breast cancer were 65.5% and 80.7%, respectively [6, 7]. Since the colorectal CSP has only been fully operational since 2019, it is too early to draw any conclusions on trends regarding this screening programme. The lowest attendance rates are found in the four large cities of the Netherlands and fall, for all three programmes, below the minimal intended rate of 70% [4]. This seems to coincide with a relatively higher incidence and related late-stage diagnoses in the same areas [14] Hence, efforts should be made to optimize current screening uptake, especially for individuals who currently do not engage in the CSPs.

General Practitioner (GP) involvement is recognized for its ability to influence screening uptake, mostly by stimulating screening participation [15–18]. Within the Netherlands, GP involvement was earlier described as beneficial for the classical, 'hard to reach', subpopulations. [13]. Thereby, the Dutch are known for placing trust in and maintaining good long-term relationships with their GPs [19]. Despite these factors, the extent of GP involvement in the CSPs remains limited, varies between the different programmes and has changed over time [13] Unexplored is what GPs think of their role(s) in the CSPs. This study aims to fill this knowledge gap by mapping the perceptions and beliefs of GPs regarding their current and future role in the Dutch CSPs. With the long-term objective in mind that GP-involvement in the CSPs could potentially boost screening attendance.

## Methods

### Study design, recruitment of respondents and interviewees, and ethical considerations

We conducted a mixed-methods sequential explanatory study using questionnaires and semi-structured interviews to gain in-depth insight into the perspectives of GPs regarding their role in the Dutch cancer screening programmes (CSPs). This explanatory study is part of an overarching study in which we are trying to identify opportunities to optimize attendance rates for the CSPs [20].

First, a survey was developed and distributed among GPs by using our Extramural LUMC Academic Network (ELAN). This is a network of GPs in the Leiden – The Hague area of the Netherlands, that aims to improve GP care in the region, including by supporting scientific research. [21] Over 100 GPs are closely linked to ELAN. These GPs were approached via a monthly newsletter between September and December 2021 (for a total of three times) and asked to fill out an online questionnaire. The invitation included background information about the study and a link to the online questionnaire. Second, for the succeeding interviews we again invited GPs via ELAN, but also activated other networks for recruiting GPs. For the interviewed GPs it was not necessary to have completed the previous questionnaire. We initially intended to purposefully select a diverse sample of interviewees within the ELAN GP-network – considering characteristics such as: sex, experience as GP, and neighbourhood (based on reported patient population characteristics) the GP was working in – however, due to time constraints and low response rates we changed to a convenience sample. The interviews were conducted partly face-to-face and partly online (i.e., video calls), based on the GP’s preference, between October and December 2022. The interviews were conducted, audio recorded and transcribed by TB, and checked by FB, VN and MC reading the transcripts.

### Questionnaire

We developed a questionnaire containing 55 questions in total, on five different topics: (I) the CSPs in the GP-practice in general, (II-IV) the CSPs at cervical, breast and CRC specifically, and (V) three open-ended questions on the (future) role of the GP within the CSPs. Questions were on how often GPs encountered the CSPs in daily practice and on their thoughts concerning the CSPs. Most questions could be answered on a five-point rating scale ranging from strongly disagree to strongly agree. To test the comprehensiveness and clarity of the questionnaire, we piloted the questions among three potential study respondents upfront. Based on their feedback, we altered a few questions with minor language adjustments. The original questionnaire was in Dutch (translated version in the Supplementary File). Aggregated outcomes of the questionnaire, which were not traceable to individual responders, served as starting points for the interviews.

### Interviews

Multiple semi-structured interviews were conducted using a thematic topic list, grounded on the outcomes of the questionnaire. Emerged topics from the questionnaire – described separately in the results section – were: (I) The current role and responsibility of GPs, (II) the informing of GPs (i.e. whether and how GPs are informed by the screening organisation, both on the patient’s screening status and screening outcomes), (III) the invitation procedures, (IV) the need for tailor-made strategies for subpopulations, and (V) suggestions for future other optimalisation of the current CSPs.

### Analyses

As this study is explanatory, we derived the primary topics from the quantitative phase and utilized the qualitative data gathered from interviews to provide context for the quantitative outcomes. In the results section of this manuscript, the study outcomes are alsopresented in this sequential order.

Data generated by the multiple-choice questions of the questionnaire are presented descriptively, using counts and percentages. IBM SPSS (version 25) was used for analysing the data. To ensure an adequate number of cases in each category for analysis, we combined and coded the responses 'agree' and 'strongly agree' as 'agreed,' while 'disagree' and 'strongly disagree' were merged and coded as 'disagreed'.

The transcripts, emerged from the interviews, were independently coded and labelled by TB and FB using a partially pre-composed code structure (open coding). Agreement on the codes was also reached between TB and FB. For each main topic, we conducted coding on the interviews to gain insights into how to interpret the quantitative data by incorporating qualitative information. The software Atlas.ti Scientific Software Development GmbH (version 7) was used for data storage, coding, and extraction of quotes for the topics. Quotes (Q) were originally in Dutch and were translated into English for this manuscript. The quotes presented in this paper were chosen based on their eloquence on a particular topic. For an overview of all quotes see Supplementary Table 1. After completing the entire study, we orally checked whether our conclusions aligned with what the interviewees thought and had wanted to convey to us; this proved to be the case.

## Results

After an online invitation of 110 GPs, a total of 46 GPs completed the online questionnaire (response rate 42%), with a mean age of 51 years (ranging from 36–68 years). Most of the respondents were female (72%) and had more than 10 years of working experience (85%). Twenty-six percent of the GPs, the largest group, were working in the greater city of The Hague. Most GPs described their population as average regarding age and educational level, and predominantly as having a Dutch cultural background (Supplementary Table 2). Subsequent five semi-structured interviews (convenience sample), ranging from 37–46 minutes, were conducted. The interviewed GPs had comparable characteristics to those of the questionnaire responders (Supplementary Table 3).

The cancer screening programmes (CSPs) were stated as an important and repeating topic in daily practice, and most GPs receive questions regarding the CSPs on a regular basis (Table 2). During the past year, 89% of the GPs received questions concerning the cervical CSP, 70% concerning the breast CSP, and 85% concerning the CRC-SP. Most questions, across all three CSPs, related to the outcomes of the screening test(s) and potential follow-up examinations, with particular emphasis on the self-sampling test for cervical CSP. GPs reported to be most familiar with the cervical CSP, regarding the objective and practice manual of the CSP, and their intended role. Only 69% of the GPs reported being familiar with their role regarding the CRC-SP, compared with 80% for the two other CSPs. Nevertheless, almost all GPs thought that their knowledge and practice policies were sufficient and accurate concerning all three CSPs. Nevertheless, the interviews revealed that GPs, on average, lack specific knowledge on various issues, including when the GP is informed and who is responsible for arranging the referral (Q3, Q21, Q49). Regarding the way GPs discuss and value the CSPs, approximately 80% of GPs indicated that they actively promote patient involvement in CSPs. The majority of GPs maintain a positive attitude toward patient participation, with 69% expressing the belief that encouraging cancer screening is always the appropriate course of action (Q8, Q16). Only 4% of the GPs occasionally discouraged patients from participating in a CSP. In the interviews it was explained that this occurred when patients struggled with extensive comorbidities, or were already involved in (other) intensive medical trajectories. More than half (57%) of the GPs indicated that they mentioned the CSPs sometimes during consultation, even without the patient explicitly asking. From the interviews, it emerged that this was usually related to certain symptoms, such as: vaginal bleeding, a breast lump, or bowel related problems. Conversely, it also occurred that talking about the CSPs served as starting point for discussing other 'intimate' topics (Q16). Sixty-four percent of the GPs agreed that educating patients on the CSPs is part of their job. A majority of the GPs (58% agreed, 16% neutral, 26% disagreed) thought that the final decision to participate in a CSP is an individual choice, and thus should primarily be left with the individual. Although GPs suggested several options to improve the current CSPs, they generally did not feel that the programmes are currently poorly arranged (Q49, Q55 Notably, during all the interviews, the current workload of GPs was repeatedly labelled as high (Q28, Q37, Q45).

**Table 2:** Quantitative outcomes questionnaire per CSP

	CC-SP	BC-SP	CRC-SP
Questions during last year	89% (*n* = 45)	70% (*n* = 46)	85% (*n* = 46)
GP familiar with			
Objectives	76% (*n* = 45)	71% (*n* = 45)	72% (*n* = 46)
Practice manual	54% (*n* = 46)	53% (*n* = 45)	54% (*n* = 46)
Role	80% (*n* = 46)	80% (*n* = 45)	69% (*n* = 45)
Sufficient knowledge GP	93% (*n* = 46)	80% (*n* = 44)	82% (*n* = 45)
Accurate practice policy	95% (*n* = 42)	N/A	N/A
In favour of inviting via GP practice	22% (*n* = 41)	17% (*n* = 41)	17% (*n* = 42)
Wanting to know who was invited	54% (*n* = 41)	39% (*n* = 41)	49% (*n* = 43)
Wanting to know who has a positive test	73% (*n* = 40)	83% (*n* = 40)	43% (*n* = 37)
Willingness to inform patients after a positive test	75% (*n* = 40)	78% (*n* = 40)	61% (*n* = 48)

*(C)SP* (Cancer) Screening Programme, *CC* Cervical Cancer, *BC* Breast Cancer, *CRC* Colorectal Cancer, *GP* General Practitioner, *N/A* not applicable

### Topic I: Current role and responsibilities of GPs

When discussing their role, the interviewees expressed satisfaction and found it to be fitting. The programmes are seen as important, and for the GPs it makes sense that they are involved, at least for a part (Q14-16). As one interviewee mentioned (Q1): *“As GPs we have to be involved in the screening programmes. The contacts resulting from engagement are eminently suiting GPs. The programmes concern cancer, which always scares patients. This is thus an opportunity for us, where we can make a difference. Patients appreciate it when we are involved, when we guide them along the way”.* More than once, the CSPs were described as part of 'indicated prevention', and thus as a task for the GP (Q4, Q6). Regarding their wish to stay involved in the CSPs, GPs indicated that they like to stay involved, and in doing so they appreciate the close relationship they have with certain patients (Q2, Q7, Q9, Q10, Q12). When addressing the topic of responsibilities, GPs concurred that they are not responsible for screening uptake (Q5, Q11). However, in the case of a positive screening outcome for an individual patient, GPs do acknowledge a sense of responsibility. This is especially evident in guiding the patient and composing referral letters (Q13) (where the latter does not apply to the CRC-SP).

### Topic II: Informing of GPs

GPs seemed to be divided regarding their preference for knowing the individuals invited by the screening organization. Approximately half of the questionnaire respondents were in favour of knowing this information, and some explicitly wrote this down in the open-ended question section. During the interviews, some stated they want to know all on attenders and non-attenders (GP IV and GP V), whereas others were more hesitant (GP I-III). This is illustrated by quotes 19, 23 and 25: *“I would like to know who did and did not participate. Now I have no clue, and therefore cannot act on it. If I knew, then I would be much better able to proactively engage with people concerning the CSPs”*, ‘versus’ quotes 18 and 20: *“I am not sure if I want to know when someone has not participated. It remains a patient’s own choice. Knowing this can be perceived as intrusive. ... Then, it may no longer feel like a free choice, but much more like coercion…”.* Several technical methods have been suggested to better inform GPs on screening attendance and outcomes; such as making use of the GP’s IT-systems (Q26), or by an opt-out based invitation system (Q27). By the latter, the interviewee meant that GPs receive information about patients' CSP attendance by default, unless patients explicitly object. In the questionnaire, 73% of the respondents indicated that they want to know who had a positive screening outcome for the cervical CSP, 83% for the breast CSP, but only 43% for the CRC-SP. As became from the interviews, the lower percentage for the CRC-SP may stem from the perception that a positive Faecal Immunochemical Test (FIT, formerly the iFOBT) is considered less serious than a positive outcome in the other two CSPs. In addition, GPs were found to be less willing to inform patients after a positive FIT outcome. Finally, certain GPs interviewed expressed concerns that being aware of individuals who did not participate in the CSPs might result in an increased workload (Q17, Q22, Q24). They believed that this knowledge would entail additional responsibilities, such as actively reaching out to those who did not attend.

### Topic III: Inviting via GP-practices

As in the past, screening-eligible people were invited via GP-practices for the cervical CSP, we questioned GPs on this topic. In the questionnaire 63% of the respondents declared they used to invite patients via their GP-practice for the cervical CSP, while 18% reported: 'unknown to me'. Only a minority (20%) of GPs currently favoured inviting patients via GP-practices. During the interviews, none of the GPs appeared to be willing to (re-)start the invitation procedures primarily via GP-practice. Indicated reasons were mostly: lack of available time, or that their time could be better spent on other things (Q29, Q31, Q34). On the other hand, GPs also realized that the involvement of GP-practices would probably lead to a higher screening uptake (Q28, Q33, Q36). A kind of 'add-on methodology' where GPs can decide, maybe in agreement with the national screening organisation, to also invite patients themselves, so in addition to the general invitation, was considered as a possible positive proposal by all the interviewees. This idea was first introduced by GP I, Q30: “*Everyone is invited by default, but on top, GPs are given a list of high-risk screening-eligible people… You could be more creative than either just the entire invitation via the screening organisation, or via GPs”.* And then later named by GP II (Q32): *“What could be done is a kind of 'add-on methodology'. So, in addition to a common basis, something extra can be done on the community-level by GP-practices. Think of a letter, or maybe even a call from the practice”.* Such a methodology seems to be in line with Q35, which addressed that screening-eligible people currently do not feel seen individually. Another, less intrusive strategy, would be to send the invitation letter on behalf of the GP, or with an envelope that states that the GP supports the CSPs (Q33, Q36).

### Topic IV: Tailor-made strategies for subpopulations/lower SES-neighbourhoods

By the GPs (I, III, V), working in more disadvantaged neighbourhoods, with a relatively lower socioeconomic status (SES), it was extensively discussed that tailor-made strategies are needed for specific subpopulations. As was stated (Q38): *“Given the complexity of participation, it is not surprising that people living in a low SES-neighbourhood and with a non-western migration background are less likely to participate. You have to do it all yourself, read it, understand it etc…”.* Several barriers were considered to be especially relevant for people living in the lower SES-neighbourhoods, such as: the lack of (health) literacy, poor education and certain taboos. Furthermore, GPs reported that people living in disadvantaged neighbourhoods often have low trust in everything related to the government (Q44). We found no clear consensus on what these tailor-made strategies should look like (Q39-44). The earlier described 'add-on methodology' however, was thought to be effective increasing screening uptake for socioeconomically disadvantaged populations, and was designated as positive by all GPs. Accurate information in several languages, and proactively approaching screening-eligible people were furthermore often mentioned as possibilities (Q39, Q40).

### Topic V: Other optimalization opportunities

Numerous other optimalization opportunities for increasing participation were suggested in the open-ended questions of the questionnaire and by the interviewed GPs. Most of the idea’s involved solutions as: making use of education videos on smartphones, pictograms, QR-codes and influencers (Q48, Q50, Q51). Furthermore, the waiting room information screen was suggested as a useful tool for informing patient on the CSPs (Q53). Despite the various technological solutions, the majority of GPs also expressed a consensus that maintaining personal contact with a GP or GP practice should still be possible (Q52). GPs noted that they do not necessarily feel that a GP is required for these interactions. Instead, there was a greater emphasis on the appropriateness of involving a (specialized) practice-based nurse (Q46). Two GPs in particular addressed the funding concerning the CSPs and prevention in general (Q45, Q47, Q57): “*… the budget for primary care will truly have to increase substantially. We … actions within the system could then be funded much more easily”.* Other suggestions involved (more) cooperation at both the regional as national level (Q56), and the training of medical students (Q58). One suggestion concerned the CRC-SP in particular. Multiple GPs observed that patients with a positive FIT are much more worried and anxious, than patients with positive outcomes at the other two CSPs. Therefore, they suggested that deeper clarification is needed on the meaning of the FIT for the public. This message should at least contain that a positive FIT, does not (immediately) equal CRC (Q54).

## Discussion

This mixed-methods study aimed to map the role of GPs in the Dutch cancer screening programmes (CSPs), indicate that the CSPs are a regular topic during consultation hours and that GPs in general have a positive attitude towards the CSPs, and towards screening participation. GPs are most often consulted regarding the cervical CSP and the CRC-SP, and most questions are related to the outcomes of the screening tests and related follow-up examinations. The current role of GPs is generally evaluated as appropriate by GPs, and they would like to remain involved in the CSPs. GPs are not in favour of inviting screening-eligible people via their practices, or taking on more logistical/organizational tasks, but are willing to empower the CSPs. GPs agreed that they want to be informed on all positive test outcomes, but there was no consensus on knowing the participation status of all, nor all screening outcomes. Several options have been proposed to improve and optimise current CSPs, especially to increase screening uptake for socioeconomically disadvantaged populations.

To our knowledge, this is the first study to map in-depth the role of the GP regarding all three Dutch CSPs, and then specifically concerning perceptions and beliefs that GPs have about their role(s) and optimalization possibilities. Most of the current literature focusses usually only on one of the CSPs and GP involvement, related to screening uptake and/or GP attitudes.. The findings of our study are consistent with these prior studies. As our findings indicate that GPs generally exhibit a positive attitude toward the CSPs, and they possess the ability to influence screening attendance rates [15–18, 22–24]. In addition, we found that GPs are aware of and willing to ensure that individuals with a potentially higher risk of developing the screening-specific tumours, who often live in relatively disadvantaged lower SES-neighbourhoods, participate in the CSPs. There is evidence in the literature that GPs are able to increase screening participation among people at higher risk, which was mostly achieved by approaching and inviting people selectively [25, 26].

GPs were found to be most familiar with the cervical CSP, which is not surprising, since current GP involvement is most prominent in this CSP [5]. GPs seemed to be especially interested in CSP aiming at breast cancer, as they were most interested in knowing who had an abnormal mammogram and were most willing to discuss positive screening outcomes with patients themselves. This is likely related to how serious positive screening outcomes are valued by GPs. Earlier research described that GPs value a positive FIT outcome much less serious, than a positive mammography outcome, [27] as was also stated by several GPs included in our study. GPs appeared to be less familiar with the CRC-SP, which is most likely related to the novelty of the programme [5]. A study focused on the CRC-SP concluded that GPs should take on a 'guidance-role' concerning possible false-positive CRC screening outcomes [28]. Responding GPs in our study explicitly stated that they like such a 'guidance-role', and do see this as a GP’s task. We therefore believe that such a guidance role of GPs could be applied to the entire portfolio of the CSPs.

Regarding our study there are certain issues which need to be reflected on. First, our questionnaire yielded a response rate of 42%, which is comparable with the results of other questionnaires searches among physicians [29]. With (online) questionnaires, there is always a potential risk of selection bias [30]. In our case, it could be that GPs who consider the CSP important participated in our study. However, as the results of the interviews align with the results of the questionnaire, we believe that we managed to minimize this risk. Second, during the interviews, we noticed that several GPs sometimes lacked parts of necessary background information to answer certain questions. For instance, most GPs assumed that they would always be informed when a patient had a positive FIT result; which is not the case (see Table 1). As described earlier, this constitutes an outcome of our study; yet it also impedes a more profound exploration of certain topics. For forthcoming studies, it could be crucial to consider that the average GP may not possess a comprehensive understanding of the organization of the CSPs. Third, during the interviews, it emerged that GPs had not always thoroughly considered their reasons for wanting certain information. For example, they regularly indicated that they wanted to know all on who had been invited, as well as on the outcomes of all screening tests. However, when we further probed into what they intended to do with this information, clear answers were not always provided. Fourth, for this study, we used a f convenience sample, due to logistical and time-related issues. Although most interviews yielded about the same answers, we cannot state that we achieved data saturation, as is often aimed for in qualitative studies [31]. Future (qualitative) studies are thus needed to clarify the above issues, which could also analyse possible differences in GP-specific characteristics related to outcomes. Lastly, as we conducted our study with GPs in (highly urbanised areas of) the Netherlands, our conclusions are primarily valid for Dutch GPs. GP involvement in the CSPs is however, not unique for the Netherlands, [15–18, 22, 24, 30, 32, 33] therefore we believe that interested readers (e.g., healthcare professionals and policymakers) from other (European) countries could also benefit from the insights gained from this study.

Based on the results of this study, we are confident that the future role of GPs can be optimised. One of the most cited concepts in the interviews was the idea of an 'add-on methodology' to increase current screening uptake, which might be particular suited for the more deprived neighbourhoods. This is in line with a more proactive, population/neighbourhood/community-oriented primary care approach and fits into the description of structured Population Health Management

 [34]. Such an 'add-on methodology' can be organised as a proactive tool, aiming to prevent adverse health events resulting from missing early screening opportunities in populations specifically at risk. A tool like this also responds to the concept of 'trust' in primary care and pays attention to people as individuals. Moreover positive endorsement can be promoted by a GP practice. Another important, and recurring issue in the interviews was the currently increasing workload of GPs [35]. In our view, the prospect of getting even busier hinders potential innovations in primary care. This phenomenon is not desirable given all the challenges in the current healthcare landscape. We would therefore advocate that new innovations to optimise current CSPs should be implemented only in close consultation with GPs.

For the nearby future, we would like to challenge the national screening organisation, together with GP-practices, to determine whether such an 'add-on methodology' can be rolled out in several neighbourhoods, and to evaluate whether this approach is indeed effective for increasing current attendance rates among screening-eligible people, ideally for those at highest cancer risks. Considering the results of this study, it would be logical to establish a pilot study in the greater city of The Hague. The hope is that if GPs are more involved in the CSPs, they can especially educate and motivate people with potentially higher pre-existing risks of developing cancer to get screened. In this regard, attention must also be given to communication from GPs to potential participants, as it is known that the way of communicating influences perceptions on the CSP [36]. In this context, consideration can also be given to shared decision-making tools, where thought should be given to what can help involve individuals who are currently not participating in the CSPs. Recent research suggests that shared decision-making tools appear to be particularly useful for people belonging to socially disadvantaged groups. A prerequisite hereby is that there is sufficient time available for the consultation [37]. Ultimately, it is most important to screen those with the highest risk of developing the screening-specific tumours.

## Conclusion

Our study indicated that the cancer screening programmes (CSPs) are a regular topic during consultation hours and that GPs judge this as a topic in which they like to stay involved. GPs are not eager to take on more logistical/organisational tasks, but are willing to positively empower the CSPs and especially targeting subpopulations at highest risk. Several suggestions emerged from our study to further optimise the CSPs. A targeted proactive primary care approach was suggested as a desirable option.

### Supplementary Information

**Supplementary Material 1.**

**Supplementary Material 2.**

**Supplementary Material 3.**

## Data Availability

The datasets generated and/or analysed during the current study are not publicly available due to the size of the data and the qualitative nature of the data, but are available in modified format from the corresponding author on reasonable request. Survey results are also available from the corresponding author upon reasonable request.

## Abbreviations

CRC: Colorectal Cancer
CSP: Cancer Screening Programme
ELAN: Extramural LUMC Academic Network
FIT: Faecal Immunochemical Test
GP: General Practitioner
RIVM: National Institute for Public Health and the Environment
SES: Socioeconomic Status
SP: Screening Programme
Q: Quotes
WHO: World Health Organization
