# Supplementary material for: Perceptions and beliefs of general practitioners on their role in the cancer screening programmes in the Netherlands: a mixed-methods study
Source: BMC Prim Care. 2024 Apr 24;25:129. doi: 10.1186/s12875-024-02394-5 (PMC11040810; doi:10.1186/s12875-024-02394-5)
Supplement: Supplementary file 1 — Supplementary Material 1. [file 12875_2024_2394_MOESM1_ESM.docx]

**Supplementary Table 1.** Quotes resulting from the interviews (n=5)

| Topic | Number | GP | Quote |
| --- | --- | --- | --- |
| Topic I | Q1 | I | As GPs we have to be involved in the screening programmes. The contacts resulting from engagement is eminently suiting GPs. The programmes are concerning cancer, which always scares patients. This is thus an opportunity for us, where we can make a difference. Patients appreciate it very much when we are involved, and when we guide them along the way. This should also be part of a GP’s natural interest. |
|  | Q2 | I | It is important that a GP personally calls if a screening outcome is showing abnormalities. For patients it is a 'bad news call', women (people) are shocked by that. I think, that we as GPs should have these kind of conversations. Thereby, it is also handy; so we can keep track of our patients. |
|  | Q3 | I | It would help, though, to have even more clarity on what is expected of you as a GP with regard to the CSPs. Especially since it changes over time. |
|  | Q4 | I | We are talking about indicated prevention, this simply is part of the GP’s job. |
|  | Q5 | I | I never really felt responsible for the CSPs, or at least not concerning the execution of the programmes. The initiative does not lie with the GP; it could only be, as maximum, a shared responsibility to meet certain targets. Then you will have to formulated a target together first; what do you want the minimum uptake to be? |
|  | Q6 | II | In my opinion indicated prevention, such as: smoking cessation, reducing obesity and cancer screening, is part of the range of tasks of a GP. This also makes sense since we know our patients and thus know on who we should focus. |
|  | Q7 | II | I want to be close to my patients. I like that, therefore I also decided to become a GP. For me it does not feel like an extra task to make an phone call regarding a positive CSP outcome. Patients really appreciate this too. It makes the work fun. So it is positive from two sides. |
|  | Q8 | III | I try to motivate patients, and if the screening outcomes return positive, then that they also participate in the follow-up tests. Most people are scared after getting a positive test-outcome. |
|  | Q9 | III | In my opinion, the CSPs are in essence not part of a GP’s job. It is fine to be indirectly involved, but this is also enough. We already have so much other things to do. I would much rather leave this to others. |
|  | Q10 | III | Regarding the guidance of patients after an oncological diagnosis it very much depends on the patient to what extent I am involved. That is really tailor-made. But very often I am involved. I also really consider that as a task for myself, and for GPs in general. |
|  | Q11 | III | I am not responsible for ensuring people to participate. There also should not be any pressure either. If there would be any pressure, GPs will immediate quit cooperating. |
|  | Q12 | IV | I call patients myself when I am informed on a positive screening outcome. A (practice-based) nurse could also do this, but it is nice to take the lead in this as GP. It is an important outcome after all. I also like doing this. As a GP, you have a relationship of trust. It is about important things and it is really nice for patients to discuss this with someone they know. That familiar face just helps. |
|  | Q13 | IV | Whether people participate or not, therefore I am certainly not responsible. That is an individual choice. But as soon as there is a positive outcome and thing needs to be done (referral, guidance etc.), it also becomes a responsibility of the GP. |
|  | Q14 | V | We cannot afford, doing nothing in terms of prevention. |
|  | Q15 | V | Of course prevention is part of a GP's job. In fact, it should be part of every consultation. |
|  | Q16 | V | I personally think discussing the CSPs is important. Mostly I recommend patients to participate in the CSPs. I also use this topic to talk about sexual health, intimate topics etc. So for me, it serves as a starting point for several issues. |
| Topic II | Q17 | I | It is nice to know whether someone has, or has not, participated in the CSPs, including the screening outcomes. However, it remains a bit of a question what to do with this information. It would take a lot of energy if GPs had to start calling/inviting/motivating everyone who did not participate in the CSPs. On the other hand, it could make sense if the programmes really prove to be very effective, in terms of decreased cancer mortality. |
|  | Q18 | I | Things are a bit complicated, as non-attender you have not been able to give consent, whether your GP is allowed to know your participation status. So regarding privacy legislation several things should be sorted out. |
|  | Q19 | II | I do think I always want to know if a patient has a positive test. Especially when you are a practice owner and know your patients well. You can use this knowledge during your consultations. The context is very important and as a GP you can act on this. |
|  | Q20 | II | I am not sure if I would want to know when someone did not participate. It remains a patient’s own choice. Knowing this can be perceived as intrusive. I think it is not right when a patient decides to not participate, the GP then gets this messages and then contacts the specific patient. Then it may no longer feel like a free choice, but much more like coercion. |
|  | Q21 | III | Strange, you would expect that we as GPs have insight in all positive outcomes. In any case, I would like to know this. Then I am also able to monitor patients and maybe discuss the outcome when that specific patient comes by. |
|  | Q22 | III | I would not necessarily want to know who did not participate. Because if I know this, then I probably have to do something with this information. |
|  | Q23 | IV | I think we would like to have insight in all screening outcomes. Thus from all who participated. This would help us during consultation and in our relation with our patients. |
|  | Q24 | IV | I would be interested to know who did not participate, but actually I have never really thought about it before. I do think it will cost a lot of energy, if we then also have to do something with this information. So if, for example, we are expected/supposed to approach all the non-attenders. The time is just not there. If there is someone who can take over, then it might be interesting. |
|  | Q25 | V | I would like to know who did and did not participate. Now I have no clue, and therefore cannot act on it. If I knew, then I would be much better able to proactively engage with people concerning the CSPs. |
|  | Q26 | V | I want there to be a pop-up in my electronic patient management system. This year patient X will be invited for this CSP. Then I will able to check if they have participated and if not, I can discuss it with them. At present, I do not think it will be too much of an added workload. I would like to give it a try. |
|  | Q27 | V | I would like to see that on all surveys, patients can very clearly tick a box to share their attendance information with their GP. Or perhaps even better, vice versa. That such consent is basically regulated, unless... |
| Topic III | Q28 | I | GPs are not waiting for more work, that is for sure. You would have to be well into the numbers to determine whether the invitation should be running via GPs (again). However, if the effect that the GPs can achieve is significant, that in, let say certain practices it saves half in terms of attendance, then, at least you should consider it. It should be a possibility if it is not running adequately in other ways. |
|  | Q29 | I | As a practice, we could start inviting potential participants ourselves (again). But then, at first it would require an estimate of how much effort this would be. You could also setup some extra assistance, which then also should be paid for. |
|  | Q30 | I | As an example: Everyone is invited by default, but on top, GPs are given a list of high-risk screening-eligible people whom you want to include in particular. You could be more creative than either just the entire invitation via the screening organisation, or via GPs. |
|  | Q31 | II | I do not think it is a good idea for GPs to start inviting. Because that is another extra task, besides, it means that we as GPs then have to take responsibility for this invitation procedures. This just has to run super smooth. We cannot have invitations not being sent, just because of some IT-failures. Or someone might not have changed their address and therefore did not receive an invitation. |
|  | Q32 | II | What could be done is a kind of 'add-on methodology'. So in addition to a common basis, something extra can be done on the community-level by GP practices. Think of a letter, or maybe even a call from the practice |
|  | Q33 | III | If you invite yourself as GP, you will probably get higher screening attendance rates. If people get a letter from an organisation they do not know, especially here in the neighbourhood, they very easily throw it away. There is a lack of trust, so to say. There is a lot of suspicion and distrust of what the government is and does. If the letter comes via the GP, or it says on the letter, "this letter is from your GP" then that will probably lead to a higher uptake. |
|  | Q34 | IV | I am not in favour of inviting myself. Right now it is well organised. We just do not have the energy and time. We already have enough things to do. |
|  | Q35 | V | People do not feel they are individually seen right now. That is also why they do not participate. This is a pity, because it could so easily be organized differently; i.e. by involving us as GPs more. We have also seen this with programmes aimed at cardiovascular risks and diabetes. If you provide individual attention, that will work. People appreciate it when they are looked after. People respond and flourish when you give them attention. |
|  | Q36 | V | I think it matters who sends the invitation letter. So whether it comes from a neutral organization/government, or via us, as GPs. This will have an effect on the screening uptake. In the past, we were involved in the invitation procedures, that worked incredibly well. It is a shame that that is no longer possible now. |
|  | Q37 | V | It is true, nowadays we have been appointed a lot of other tasks. Before, it was easy to be involved in the CSPs, but maybe now not anymore. This is also a political choice, what do we as a society want a GP to do? In addition, GPs are current busy because of the 'Purple Crocodile'. If only we could get rid of that, we would have time again to tackle really important issues. There is a desire for GPs to work more on prevention, look also at the Integral Prevention Agreement, but now it is hardly doable for us. |
| Topic IV | Q38 | I | Given the complexity of participation, it is not surprising that people living in a low SES-neighbourhood and with a non-western migration background are less likely to participate. You have to do it all yourself, read it, understand it etc. You may wonder whether sufficient instructions are provided. There has been very little attention to enlighten this problem. |
|  | Q39 | II | Information in other languages is essential; but, I think it already exists. This should be included with the invitation(s). |
|  | Q40 | II | You could choose to go more into the neighbourhoods, to talk with people, and to activate peers more. Only of course, if low attendance is really perceived as a problem. |
|  | Q41 | III | There is not just a silver bullet, you will have to aim for different things. It often starts with proper education. In addition, there are probably also many other barriers that need to be addressed. |
|  | Q42 | III | In our neighbourhood, there is a curious paradox. On the one hand we see people who are very carcinophobic and hypochondriac, yet on the other hand, they seldomly participate in the CSPs. As GPs, we could respond to that quite well, if we were better engaged. Better screening uptake is in all our interests. |
|  | Q43 | IV | I do not believe anything has to change with respect to the invitation letter or procedure. I cannot remember a patient consulting me on these matters. |
|  | Q44 | V | In this neighbourhood, there is a distrust of everything which has to do with the government. People here also think: “government you have nothing to do with my 'intimate' health”. Those people then do not participate. I could really act on this as a GP. For many people here in the neighbourhood, the GP is still quite important. It matters what the doctor says. There are also people who do not participate because they do not like the tests, or because they are afraid they will not perform them in the right manner. I could really respond to this kind of barriers/believes. |
| Topic V | Q45 | I | It would show political decisiveness to ensure that you can get by as a GP with a practice of, let say, 1.200 patients. Then, you will have time to do a lot of things and then these kinds of preventive tasks can be added much more easily. But then the budget for primary care will really have to increase substantially. We do not need to earn more as GPs, but actions within the system could then be funded much more easily. |
|  | Q46 | I | Within the practice, you could also appoint an assistant to specifically deal with the CSPs. This person could then answer questions about the CPS, perform Pap-smears, etc. Instruction videos in different languages would help too. However, the option to come to the practice, and to speak to someone should always remain possible. |
|  | Q47 | I | The GP is an easily accessible healthcare professional for a lot of people, and that is nice too. As a GP, you should also be able to continue like this, you should have time do provide these contacts. If there is a bit of extra funding for counselling potential participants, that would be really nice and would fit within current primary care. |
|  | Q48 | I | These days, I believe more and more in the possibilities of technology. Everyone has a smartphone. Everyone can watch films on it. This opens endless possibilities. More thought should be given to this. |
|  | Q49 | II | Actually, I do not think it is badly arranged now. Also the amount of GP involvement seems appropriate. What is however remarkable is the differences between the three CSPs. Why cannot just the screening organisation always make the referral, for example. Why do we as GPs still have to sit in between? |
|  | Q50 | II | I think language is often way too difficult. Language in itself can be a big problem. Written language is for many people difficult. There is a reason why 'thuisarts.nl' already has lots of videos. Besides, you should really use pictograms; and QR-codes for quick access to videos. |
|  | Q51 | II | Influencers on social media really make a differences these days, why not involve them? |
|  | Q52 | II | I think there are a lot of people who would like to talk with a healthcare professional about participating in the CSPs. GP practices would be a good place for that. It is often not just about facts and figures, but very often about trust. That is precisely where the GP (practices) can facilitate. |
|  | Q53 | III | Where you could do this in the GP's waiting room, by making use of the waiting room screen. That is an excellent place for education. Short, powerful, clear, straightforward, that works. We have had waiting room videos for years and really noticed that people learn something via this screen. People do need knowledge, but you have to really tailor it. The waiting room is pre-eminently a place where people can absorb medical information. |
|  | Q54 | III | Regarding the CRC-SP. I wonder if it is sufficiently clear to patients that this is not a test directly for cancer, but much more for its precursors. I would like people to be less shocked by the outcome. Nowadays, people are instantly worried they have cancer. |
|  | Q55 | IV | For now, most things are just fine. So then we should not want to change much. I am satisfied with how things are arranged. |
|  | Q56 | V | What I miss is cooperation. Everyone is always talking about this word. Also for the screening on cancer, it would help if healthcare providers and organizations cooperate. GPs, community centres, municipal health services, everyone is doing something, but not as a whole. We are working alongside each other. They/we are all little islands. Everyone is "helping", but who is really doing something? Where does the patient really benefit from in the end?  In addition, we as GPs are really not valued properly by the current politics/government. We could really help, but are ignored. People will participate if we as GPs ask them to. In the process, this also undermines the credibility of the entire healthcare system. |
|  | Q57 | V | I would opt that health insurers collectively put 10% into a fund. This money could then be used to set up nationwide prevention projects. |
|  | Q58 | V | Finally, I really hope that we will educate the new medical students differently. Teach them about prevention. |

GP= General Practitioner, (C)SP= (Cancer) Screening Programme

**Supplementary Table 2.** Characteristics of the questionnaire respondents (n=46)

|  |  | n | % |
| --- | --- | --- | --- |
| Age (years) | Mean: 51 (min-max: 36-68) | 46 |  |
| Contractual hours | Mean: 37 (min-max: 20-60) | 45 |  |
|  |  |  |  |
| Sex | Female | 33 | 72 |
|  | Male | 13 | 28 |
| Experience as GP (years) | 0-2 | 1 | 2 |
|  | 3-5 | 2 | 4 |
|  | 6-10 | 4 | 9 |
|  | 10-19 | 20 | 44 |
|  | ≥20 | 19 | 41 |
| Location of practice (city/village) | The Hague | 12 | 26 |
|  | Noordwijk/Leidschendam | 10 | 22 |
|  | Alphen aan de Rijn | 7 | 15 |
|  | Leiden | 6 | 13 |
|  | Delft | 5 | 11 |
|  | Zoetermeer | 4 | 9 |
|  | Hoofddorp | 2 | 4 |
| Patient population (description) |  |  |  |
| Age-range | Old (≥65 years) overrepresented | 9 | 20 |
|  | Average distribution (all ages) | 31 | 69 |
|  | Young (≤35 years old) overrepresented | 5 | 11 |
| Education | Higher education (university of applied sciences) overrepresented | 8 | 18 |
|  | Average distribution | 28 | 62 |
|  | Lower education (≤secondary vocational education) overrepresented | 9 | 20 |
| Cultural background* | Predominantly Dutch | 32 | 74 |
|  | Predominantly from Western | 4 | 9 |
|  | Predominantly from non-Western | 7 | 16 |

GP= General Practitioner
*for definition see the survey attached as supplementary file (page 12)

**Supplementary Table 3.** Characteristics of the interviewed respondents (n=5)

|  |  | n | % |
| --- | --- | --- | --- |
| Sex | Female | 3 | 60 |
|  | Male | 2 | 40 |
| Experience as GP (years) | 0-5 | 1 | 20 |
|  | 6-19 | 1 | 20 |
|  | ≥20 | 3 | 60 |
| Patient population (description) |  |  |  |
| Age | Elderly (≥65 years) overrepresented | 1 | 20 |
|  | Average distribution | 3 | 60 |
|  | Young people (≤35 years old) overrepresented | 1 | 20 |
| Education | Higher education (university of applied sciences) overrepresented | 1 | 20 |
|  | Average distribution | 3 | 60 |
|  | Lower education (≤secondary vocational education) overrepresented | 1 | 20 |
| Cultural background* | Predominantly Dutch | 3 | 60 |
|  | Predominantly from Western | 0 | 0 |
|  | Predominantly from non-Western | 2 | 40 |

GP= General Practitioner

*for definition see the survey attached as supplementary file (page 12)
