# Supplementary material for: Perceptions and beliefs of general practitioners on their role in the cancer screening programmes in the Netherlands: a mixed-methods study
Source: BMC Prim Care. 2024 Apr 24;25:129. doi: 10.1186/s12875-024-02394-5 (PMC11040810; doi:10.1186/s12875-024-02394-5)
Supplement: Supplementary file 2 — Supplementary Material 2. [file 12875_2024_2394_MOESM2_ESM.docx]

﻿**The general practitioner and the population-based cancer screening programmes**

On experiences, wishes & ideas

Dear general practitioner, dear colleague,

The Health Campus The Hague is investigating how the current population-based cancer screening programs (CSPs) can be optimised. This because it appears that fewer and fewer people are participating in the CSPs.^1-3^ For information on the overarching study, see the website: [Screening](https://healthcampusdenhaag.nl/nl/project/screening-the-city/) the CITY

As a general practitioner you currently have varying tasks regarding the CSPs aiming at cervical, breast and colorectal cancer. We would like to ask you some questions about these different tasks. In addition, we would like to know whether you feel that certain aspects should be changed when it comes to your role as a GP regarding the CSPs.

We developed a short questionnaire and would like you to fill it out. Within 10-15 minutes you are able to share your experiences, wishes & ideas with us. Naturally, the information will be treated with confidentially and processed anonymously. Afterwards, we will publish the results on our website and use them for a scientific manuscript. We hope you are willing to fill out the questionnaire. As you will understand, the more completed questionnaires, the better the results will reflect on the collective thinking.

Thank you in advance for your cooperation.

Thom Bongaerts

GP trainee, PhD candidate

Health Campus The Hague

[t.h.g.bongaerts@lumc.nl](mailto:t.h.g.bongaerts@lumc.nl)

Also on behalf of the other members of the research team:

Mattijs Numans, Onno Guicherit, Frederike Büchner, Vera Nierkens & Matty Crone

1. Rijksinstituut voor Volksgezondheid en Milieu. Landelijke Evaluatie & Monitoring Bevolkingsonderzoeken. <https://www.rivm.nl/>
2. Bevolkingsonderzoek Zuid-West. Jaarverslag 2019. <https://www.bevolkingsonderzoeknederland.nl/media/1442/jaarverslag-2019_def.pdf>
3. Bevolkingsonderzoek Midden-West. Jaarverslag 2019. <https://www.bevolkingsonderzoeknederland.nl/media/1404/126-200005-jaarverslag-2019-def_hr.pdf>**List of abbreviations**

| CSP | Cancer screening programme |
| --- | --- |
| BC-SP | Cancer screening programme aiming at breast cancer |
| CC-SP | Cancer screening programme aiming at cervical cancer |
| CRC-SP | Cancer screening programme aiming at colorectal cancer |
| FIT | Faecal Immunochemical Test (screening test CRC-SP) |
| GP | General Practitioner |
| hrHPV | High risk human papillomavirus |
| NHG | Dutch College of General Practitioners |
| Pap-test | Papanicolaou test (screening test CC-SP) |

Below are a number of statements and questions. Please choose the answer most applicable to your situation in each case. We would like you to complete all statements and questions. Comments and remarks can be made on the last page.

1. **The CSPs in the general practice**

*Following are a number of statements and questions about to which extent you deal with the cancer screening programmes (CSPs) on a daily basis. In each case, please choose the answer that best suits your situation.*

1. Patients come to the GP-practice (to me as GP and/or to the practice assistants) with questions about the CSPs.
   ⃝ strongly disagree
   ⃝ disagree
   ⃝ neutral
   ⃝ agree
   ⃝ strongly agree
2. The questions I get about the CSPs are (multiple answers possible):

⃝ mostly on the CSP aimed at cervical cancer (CC)
⃝ mostly on the CSP aimed at breast cancer (BC)
⃝ mostly on the CSP aimed at colorectal cancer (CRC)
⃝ not applicable; I don't get any questions about the CSPs

1. In the past year, have you encouraged patients to participate in the CSPs?

⃝ yes

⃝ no

1. In the past year, have you advised patients against participating in the CSPs?

⃝ yes

⃝ no

1. Do you ever bring up the CSPs without a patient explicitly asking about these programmes?

⃝ yes

⃝ no

*Following are a number of statements on the several tasks you have as a GP. Please choose the answer that best suits you.*

1. I think providing information about the CSPs is part of my job as GP.
   ⃝ strongly disagree
   ⃝ disagree
   ⃝ neutral
   ⃝ agree
   ⃝ strongly agree
2. I think I should encourage participation in the CSPs.
   ⃝ strongly disagree
   ⃝ disagree
   ⃝ neutral
   ⃝ agree
   ⃝ strongly agree
3. I feel that I should leave the choice to participate in the CSPs mainly with the patient.
   ⃝ strongly disagree
   ⃝ disagree
   ⃝ neutral
   ⃝ agree
   ⃝ strongly agree
4. I feel I should only discuss the CSPs when the patient has specific questions regarding the screening programmes.
   ⃝ strongly disagree
   ⃝ disagree
   ⃝ neutral
   ⃝ agree
   ⃝ strongly agree
5. **Specific questions about the CSP aiming at cervical cancer**

*Following questions concern your role and that of the practice assistant(s), regarding the CSP aiming at cervical cancer (CC-SP). In each case, please choose the answer that best suits you.*

1. In the past year, have you (or any of your practice assistants) had any questions about the CSP aiming at CC?

⃝ yes

⃝ no; you can proceed to question 3

1. What were the questions about (multiple answers possible):

⃝ the invitation

⃝ participation in the CSP

⃝ the risk of developing cervical cancer

⃝ the outcome of the (screening) test

⃝ the self-test

⃝ follow-up examinations

⃝ participation at the follow-up examinations

*Following statements are about your experiences with the CSP aiming at cervical cancer (CC-SP). Please choose the answer that best suits you.*

1. I am well informed about the content and objectives of the CC-SP.
   ⃝ strongly disagree
   ⃝ disagree
   ⃝ neutral
   ⃝ agree
   ⃝ strongly agree
2. I am aware of the NHG practice manual on the CC-SP.
   ⃝ strongly disagree
   ⃝ disagree
   ⃝ neutral
   ⃝ agree
   ⃝ strongly agree
3. I know what my role is regarding to the CC-SP.
   ⃝ strongly disagree
   ⃝ disagree
   ⃝ neutral
   ⃝ agree
   ⃝ strongly agree
4. I have sufficient knowledge to explain about the CC-SP.
   ⃝ strongly disagree
   ⃝ disagree
   ⃝ neutral
   ⃝ agree
   ⃝ strongly agree
5. In the practice where I work, we (GPs and practice assistants) know how to perform PAP-tests according to the CC-SP guidelines.
   ⃝ strongly disagree
   ⃝ disagree
   ⃝ neutral
   ⃝ agree
   ⃝ strongly agree

*Following questions and statements are about your vision of the CSP aiming at cervical cancer (CC-SP). Please choose the answer that best suits you.*

*In the past, invitations to participate in the CC-SP were sent via GP practices. The national participation rate was at the time higher.*

1. Were women in your practice actively invited to participate in the CC-SP in the past?

⃝ yes

⃝ no; you can proceed to question 10

⃝ unknown to me; you can proceed to question 10

1. Since women are no longer invited via GP practices, I noticed that fewer women are participating in the CC-SP.
   ⃝ strongly disagree
   ⃝ disagree
   ⃝ neutral
   ⃝ agree
   ⃝ strongly agree
2. I (again) would like to have the possibility to invite women for the CC-SP.
   ⃝ strongly disagree
   ⃝ disagree
   ⃝ neutral
   ⃝ agree
   ⃝ strongly agree
3. I want to know which of 'my' patients were invited for the CC-SP.
   ⃝ strongly disagree
   ⃝ disagree
   ⃝ neutral
   ⃝ agree
   ⃝ strongly agree

*For the CC-SP, the possibility of using the hrHPV (high-risk human papillomavirus) self-test exists since 2017. As a result, it is no longer necessary for women to have a smear test taken at the GP practice, but women can independently test for hrHPV. The GP does not receive the outcomes of a self-test .This is in the context of privacy legislation. If hrHPV is found with the self-test, a woman is advised to have a smear taken at the GP practice. This smear is then cytologically assessed.*

1. As a GP, I always want to know if a patient has taken a self-test as part of the CC-SP.
   ⃝ strongly disagree
   ⃝ disagree
   ⃝ neutral
   ⃝ agree
   ⃝ strongly agree
2. When women receive a positive screening outcome, I want to be able to inform them myself.
   ⃝ strongly disagree
   ⃝ disagree
   ⃝ neutral
   ⃝ agree
   ⃝ strongly agree

*Depending on the outcomes of the screening test, the GP is still involved by partaking a control smear after 6 months, or by referring the women to a gynaecologist for follow-up examinations.*

*The GP will always be informed about outcomes emerging from the follow-up examination(s).*

1. **Specific questions about the CPS aiming at breast cancer**

*Following questions concern your role regarding the CSP aimed at breast cancer (BC-SP). In each case, please choose the answer that best suits you.*

1. Have you had any questions about the BC-SP in the past year?

⃝ yes

⃝ no; you can proceed to question 3

1. What were the questions about (multiple answers possible):

⃝ the invitation

⃝ the invitation interval (actual since Covid-19)

⃝ participation in the CSP

⃝ the risk of developing breast cancer

⃝ the outcome of the (screening) test

⃝ follow-up examinations

⃝ participation at the follow-up examinations

*Following statements are about your experiences with the CSP aiming at breast cancer (BC-SP). Please choose the answer that best suits you.*

1. I am well informed about the content and objectives of the BC-SP.
   ⃝ strongly disagree
   ⃝ disagree
   ⃝ neutral
   ⃝ agree
   ⃝ strongly agree
2. I am aware of the NHG practice manuals on the BC-SP.
   ⃝ strongly disagree
   ⃝ disagree
   ⃝ neutral
   ⃝ agree
   ⃝ strongly agree
3. I know what my role is regarding the BC-SP.
   ⃝ strongly disagree
   ⃝ disagree
   ⃝ neutral
   ⃝ agree
   ⃝ strongly agree
4. I have sufficient knowledge to explain about the BC-SP.
   ⃝ strongly disagree
   ⃝ disagree
   ⃝ neutral
   ⃝ agree
   ⃝ strongly agree

*Following statements are about your vision of the future regarding the CSP aiming at breast cancer (BC-SP). Please choose the answer that best suits you.*

1. I want to know which women from my practice, have been invited for the BC-SP.
   ⃝ strongly disagree
   ⃝ disagree
   ⃝ neutral
   ⃝ agree
   ⃝ strongly agree
2. I want to be able to invite women for the BC-SP.
   ⃝ strongly disagree
   ⃝ disagree
   ⃝ neutral
   ⃝ agree
   ⃝ strongly agree

*As GP, you will be involved in the BC-SP when follow-up examinations are needed as a result of the mammograms. As GP you need to refer the specific women to a hospital for further analysis. This may be because the X-rays are not conclusive, or if the X-rays show abnormalities.*

1. As a GP, I always want to know if a patient has had a mammogram as part of the BC-SP.
   ⃝ strongly disagree
   ⃝ disagree
   ⃝ neutral
   ⃝ agree
   ⃝ strongly agree
2. When women gets an abnormal screening outcome, I want to be able to inform them myself.
   ⃝ strongly disagree
   ⃝ disagree
   ⃝ neutral
   ⃝ agree
   ⃝ strongly agree

*The GP will always be informed on the outcomes following the follow-up examination(s).*

1. **Specific questions about the CSP aiming at colorectal cancer**

*The following questions are about your role at the CSP aiming at colorectal cancer (CRC-SP). Please choose the answer that best suits you.*

1. Have you had any questions about the CRC-SP in the past year?

⃝ yes

⃝ no; you can proceed to question 3

1. What were the questions about (multiple answers possible):

⃝ the invitation

⃝ participation in the CSP

⃝ the risk of developing colorectal cancer

⃝ the outcome of the (screening) test

⃝ follow-up examinations

⃝ participation at the follow-up examinations

*The following statements are about your experiences with the CSP for colorectal cancer (CRC-SP). Please choose the answer that best suits you.*

1. I am well informed about the content and objectives of the CRC-SP.
   ⃝ strongly disagree
   ⃝ disagree
   ⃝ neutral
   ⃝ agree
   ⃝ strongly agree
2. I am aware of the NHG practice manuals on the CRC-SP.
   ⃝ strongly disagree
   ⃝ disagree
   ⃝ neutral
   ⃝ agree
   ⃝ strongly agree
3. I know what my role is regarding the CRC-SP.
   ⃝ strongly disagree
   ⃝ disagree
   ⃝ neutral
   ⃝ agree
   ⃝ strongly agree
4. I have sufficient knowledge to explain about the CRC-SP.
   ⃝ strongly disagree
   ⃝ disagree
   ⃝ neutral
   ⃝ agree
   ⃝ strongly agree

*Following question and statements are about your vision on the future of the CSP aimed at colorectal cancer (CRC-SP).Please choose the answer that best suits you.*

1. I would like to know who participated in the CRC-SP.
   ⃝ strongly disagree
   ⃝ disagree
   ⃝ neutral
   ⃝ agree
   ⃝ strongly agree
2. I would like to be able to invite patients for the CRC-SP myself.
   ⃝ strongly disagree
   ⃝ disagree
   ⃝ neutral
   ⃝ agree
   ⃝ strongly agree

*Since January 2017, GPs are no longer automatically notified on the outcomes of the FIT; the primary screening test for the CRC-SP. This is in the context of privacy legislation. Participants must give explicit consent for sharing information regarding the FIT. In case of a positive FIT outcome, a patient receive an appointment for follow-up testing by the screening organization. Patients are advised to contact their GP if they receive a positive FIT outcome.*

1. Were you aware of this change?

⃝ yes
⃝ no

1. As a GP, I always want to know whether a patient has submitted an FIT as part of the CRC-SP.
   ⃝ strongly disagree
   ⃝ disagree
   ⃝ neutral
   ⃝ agree
   ⃝ strongly agree
2. As a GP, I always want to know if a patient had a positive FIT.
   ⃝ strongly disagree
   ⃝ disagree
   ⃝ neutral
   ⃝ agree
   ⃝ strongly agree
3. When patients from my practice receive a positive screening outcome, I want to be able to inform them myself.
   ⃝ strongly disagree
   ⃝ disagree
   ⃝ neutral
   ⃝ agree
   ⃝ strongly agree

*The GP will always be informed about outcomes following the follow-up examination(s).***Descriptive characteristics**

Finally, a few questions about you as a GP, and the place where you work.

1. What is your year of birth?

|  |
| --- |

1. What is your gender?
   ⃝ Male
   ⃝ Female
2. What kind of professional appointment do you have?

⃝ Practice owner

⃝ Employed GP (at a permanent practice)

⃝ Acting general practitioner

1. What are the first 2 digits of the zip code where you work as a GP? (if you are an acting GP, please enter the postcode of the practice where you most often work)

|  |
| --- |

1. How many years of work experience do you have as a GP?

⃝ 0-2

⃝ 3-5
⃝ 6-10

⃝ 10-20

⃝ 20+

1. On average, how many hours per week do you work (contract hours)?

|  |
| --- |

1. How would you describe your patient population with respect to age distribution?

⃝ elderly (≥65 years) overrepresented
⃝ average distribution

⃝ young people (≤35 years old) overrepresented

1. How would you describe your patient population with respect to level of education?

⃝ higher education (university of applied sciences) overrepresented

⃝ average distribution

⃝ lower education (≤Secondary vocational education) overrepresented

1. How would you describe your patient population with respect to cultural background?

⃝ predominantly from a Dutch background

⃝ predominantly from a Western-migration background. (Countries in Europe, North America, Oceania, Indonesia and Japan; excluding Turkey)

⃝ predominantly from non-Western migration backgrounds. (countries in Africa, Latin America and Asia (excluding Indonesia and Japan) or Turkey)

1. **Open questions**
2. Do you have any general comments regarding this questionnaire?

|  |
| --- |

1. Do you think GPs should have a role regarding the CSPs? If so, how do you think that role should look like?

|  |
| --- |

1. Are there any other things you would like to add which have a relation with the CSPs, and/or could possibly be of additional value to our research?

|  |
| --- |

Thank you very much for your participation!
