# Supplementary material for: Perceptions and beliefs of general practitioners on their role in the cancer screening programmes in the Netherlands: a mixed-methods study
Source: BMC Prim Care. 2024 Apr 24;25:129. doi: 10.1186/s12875-024-02394-5 (PMC11040810; doi:10.1186/s12875-024-02394-5)
Supplement: Supplementary file 3 — Supplementary Material 3. [file 12875_2024_2394_MOESM3_ESM.pdf]

Checklist of Mixed Methods Elements in a Submission to Advance the Methodology of Mixed Methods Research

Manuscript title:	Yes	No	Page number (if not applicable, indicate NA)
**Title**			
1. Does the title directly indicate or sufficiently allude to the methodological contribution of the article?	☑	☐	1
**Abstract**			
2. Does the abstract include an explicit statement about a methodological challenge or issue in the field that will be addressed in the article?	☑	☐	2
3. Does the abstract indicate the methodological/theoretical contribution of the article to the field of mixed methods research?	☐	☒	2
**Main text of the article**			
4. Does the article have a clear writing style with sufficient headers and sub-headers such that the reader can readily follow the flow and argumentation?	☑	☐	Throughout the manuscript
5. Does the text in the background reiterate and expand upon the methodological challenge or issue as identified in the abstract?	☑	☐	4-6
6. Does the background contain a rigorous review and citations of relevant and recent mixed methods literature to support examining the methodological aim?	☐	☒	In general, not purely on mixed methods
7. Does the background include an explicit methodological aim?	☑	☐	6
8. Does the background contain an explication of the article's structure and methodological points that will be addressed?	☐	☒	In methods section
9. In the body of article, are each of the methodological points identified in #8 addressed persuasively in the order specified?	☑	☐	9-14
10. Does the article include a strategy to convey the overall complexity of the topic or study phenomenon such as a figure or illustration?	☑	☐	Tables and Supp Tables
11. In the discussion, are the explicit points made in #8 synthesized together to logically support the overarching methodological aim?	☑	☐	15
12. Does the discussion section include a specific subsection "Contribution to the Field of Mixed Methods Research" that reviews the points made and extant literature to articulate the articles novel contribution(s) to mixed methods?	☐	☒	Article not about the method, but uses it
13. Does the article have a discussion of the methodological limitations?	☑	☐	16
14. Does the discussion section include recommendations for future mixed methods inquiry based on the paper's unique contribution or limitations?	☑	☐	~~16~~
15. Have the references been cited according to the current American Psychological Association style?	☑	☐	
**Additional elements for empirical methodological articles only**			
16. Does the background of the article include explicit statements of both the methodological aim and purpose of the empirical study separately?	☐	☐	
17. Does the description of the methods include sufficient detail about the procedures used and present these in a logical order?	☐	☐	
18. Does the submission include a procedural diagram of the data collection and analysis procedures as a figure?	☐	☐	
19. Does the submission include a table, matrix or visual structure, e.g., joint display, to illustrate integration and interpretation of the qualitative and quantitative findings?	☐	☐	
20. Does the discussion articulate how the use of a mixed methods approach advanced a greater understanding of the substantive topic compared to using a monomethod approach?	☐	☐	

.(Adapted from Fetters and Freshwater (2015a) and Fetters and Molina-Azorin (2019
